# Activation of transient receptor potential vanilloid 4 induces apoptosis in hippocampus through downregulating PI3K/Akt and upregulating p38 MAPK signaling pathways

**DOI:** 10.1038/cddis.2015.146

**Published:** 2015-06-04

**Authors:** P Jie, Z Hong, Y Tian, Y Li, L Lin, L Zhou, Y Du, L Chen, L Chen

**Affiliations:** 1 Department of Physiology, Nanjing Medical University, Nanjing, PR China; 2 Research Center of Ion Channelopathy, Institute of Cardiology, Union Hospital, Tongji Medical College, Huazhong University of Science and Technology, Wuhan, PR China

## Abstract

Transient receptor potential vanilloid 4 (TRPV4) is a calcium-permeable cation channel that is sensitive to cell swelling, arachidonic acid and its metabolites, epoxyeicosatrienoic acids, which are associated with cerebral ischemia. The activation of TRPV4 induces cytotoxicity in many types of cells, accompanied by an increase in the intracellular free calcium concentration. TRPV4 activation modulates the mitogen-activated protein kinase (MAPK) and phosphatidyl inositol 3 kinase (PI3K)/ protein kinase B (Akt) signaling pathways that regulate cell death and survival. Herein, we examined TRPV4-induced neuronal apoptosis by intracerebroventricular (ICV) injection of a TRPV4 agonist (GSK1016790A) and assessed its involvement in cerebral ischemic injury. ICV injection of GSK1016790A dose-dependently induced apoptosis in the mouse hippocampi (GSK-injected mice). The protein level of phosphorylated p38 MAPK (p-p38 MAPK) was markedly increased and that of phosphorylated c-Jun N-terminal protein kinase (p-JNK) was virtually unchanged. TRPV4 activation also decreased Bcl-2/Bax protein ratio and increased the cleaved caspase-3 protein level, and these effects were blocked by a PI3K agonist and a p38 MAPK antagonist, but were unaffected by a JNK antagonist. ICV injection of the TRPV4 antagonist HC-067047 reduced brain infarction after reperfusion for 48 h in mice with middle cerebral artery occlusion (MCAO). In addition, HC-067047 treatment attenuated the decrease in the phosphorylated Akt protein level and the increase in p-p38 MAPK protein level at 48 h after MCAO, while the increase in p-JNK protein level remained unchanged. Finally, the decreased Bcl-2/Bax protein ratio and the increased cleaved caspase-3 protein level at 48 h after MCAO were markedly attenuated by HC-067047. We conclude that activation of TRPV4 induces apoptosis by downregulating PI3K/Akt and upregulating p38 MAPK signaling pathways, which is involved in cerebral ischemic injury.

Transient receptor potential vanilloid 4 (TRPV4), a member of the transient receptor potential (TRP) superfamily, is permeable to calcium (Ca^2+^).^1^ TRPV4 was first described as a cellular osmotic sensor that detects hypotonic stimulation, and it has now been proven to be activated by multiple stimuli, including mild heat, mechanical stimulation, arachidonic acid (AA) and its metabolites, and exogenous chemical ligands.^2^ TRPV4 is widely expressed in the nervous system and other tissues, including the lungs, bladder and skin.^1^ In the central nervous system, TRPV4 is present in neurons and glial cells.^3, 4^ It mediates infrasound- and beta amyloid peptide-induced neuronal impairment, accompanied by an increase in the intracellular free calcium concentration ([Ca^2+^]_i_).^5, 6^ Application of a TRPV4 agonist dose-dependently induces hippocampal neuronal death *in vivo*.^7^ Additionally, a gain-of-function mutant of TRPV4 has been shown to augment Ca^2+^ entry and decrease cell viability in transfected HEK293 cells.^8^ TRPV4 can be activated by cell swelling-induced mechanical stimulation and metabolites of AA that are always associated with cerebral ischemia. The protein level of TRPV4 has been reported to increase with ongoing reperfusion in a mouse model of middle cerebral artery occlusion (MCAO).^7^ Therefore, the over- or hyper-activation of TRPV4 is likely during cerebral ischemia-reperfusion. Blocking of TRPV4 has been shown to exert neuroprotective effects against cerebral ischemic injury in both *in vitro* and *in vivo* studies.^7, 9, 10, 11^ Targeting of TRPV4 is attracting more and more attention in the treatment of cerebral ischemia.

Cell apoptosis, which is one of the major causes of cerebral ischemic injury, becomes prominent after reperfusion for 24–72 h.^12^ It has been reported that excessive Ca^2+^ entry through TRPV4 leads to apoptosis in mouse retinal ganglion cells, which may be due to the activation of Ca^2+^-dependent pro-apoptotic signaling pathways.^13^ Mitogen-activated protein kinase (MAPK) signaling pathways that are involved in cerebral ischemic injury have important roles in regulating cell death and survival through signal translocation pathways related to apoptosis.^14^ The activation of phosphatidyl inositol 3-kinase (PI3K)/protein kinase B (Akt) signaling has been reported to inhibit caspase-dependent apoptosis in cultured neurons and a mouse model of Alzheimer's disease.^15, 16, 17^ Activation of TRPV4 can modulate MAPK and PI3K/Akt signaling pathways in different types of cells.^7, 18^ In this study, we first assessed the effect of TRPV4 activation on neuronal apoptosis in the hippocampus and then explored the mechanisms underlying TRPV4 action. Finally, we examined the involvement of TRPV4-induced apoptosis in MCAO in mice.

## Results

### Effect of TRPV4 agonist on apoptosis in hippocampus

**Figure 1:**
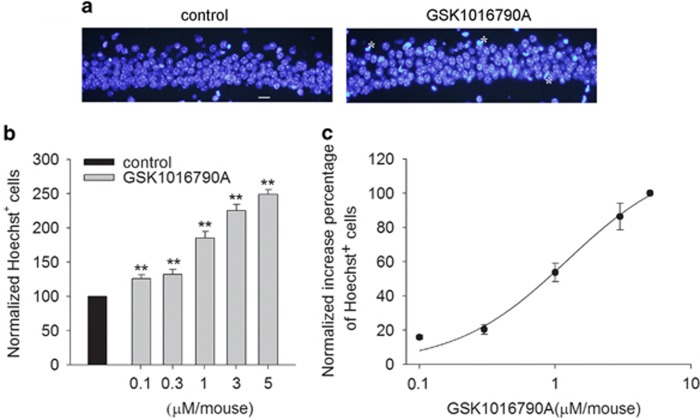
TRPV4-induced apoptosis in hippocampus. (**a**) Hoechst staining shows that ICV injection of a TRPV4 agonist GSK1016790A (1 *μ*M/mouse) induced apoptosis in the hippocampal CA1 area. Scale bar=50 *μ*M. (**b**) The bar graph shows the numbers of Hoechst^+^ cells in the hippocampal CA1 area in the presence of different doses of GSK1016790A. **P<0.01 *versus* control mice. (**c**) The dose-dependent curve of GSK1016790A-induced apoptosis in the hippocampal CA1 area

In this study, we first examined whether the activation of TRPV4 induces apoptosis in the hippocampus by administering intracerebroventricular (ICV) injections of different doses of the TRPV4 agonist GSK1016790A (GSK-injected mice). The number of Hoechst^+^ cells in the hippocampal CA1 area was 4.85±2.17 /mm in the control group, which is consistent with a previous report.^19^ Figure 1a shows that more Hoechst^+^ cells were detected after the mice were injected with GSK1016790A (1 *μ*M/mouse) compared with the control value. Moreover, at doses ranging from 0.1 *μ*M/mouse to 5 *μ*M/mouse, GSK1016790A-induced apoptosis was dose dependent, with EC_50_ values being 1.21±0.24 *μ*M/mouse (Figures 1b and c). These results provide *in vivo* evidence that the over-activation of TRPV4 may result in apoptosis in the hippocampus. In the presence of 1 *μ*M/mouse GSK1016790A, the number of Hoechst^+^ cells in the hippocampal CA1 area was increased by 80.67±1.16% (*P*<0.01), and this dose was used in the following experiments.

### Effect of TRPV4 agonist on the expression of apoptosis-related signaling pathways and apoptosis-related proteins

**Figure 2:**
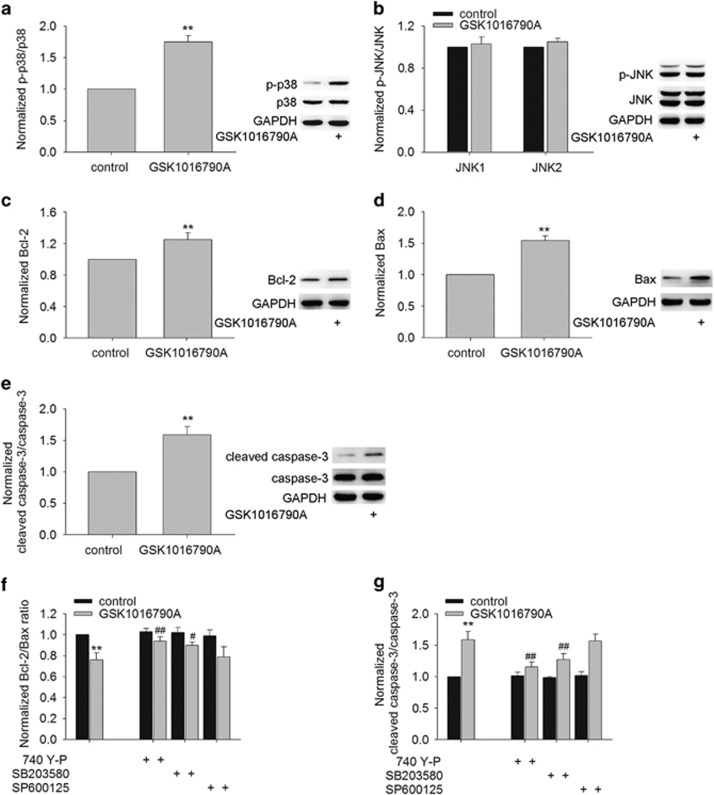
TRPV4-induced modulations of apoptosis-related signaling pathways and apoptosis-related proteins. (**a**–**e**) Western blot analysis showing the protein levels of p-38 MAPK (**a**), p-JNK (**b**), Bcl-2 (**c**), Bax (**d**) and the cleaved caspase-3 (**e**) in the hippocampi of mice injected with vehicle (control mice) and GSK1016790A, respectively. (**f** and **g**) TRPV4-induced decrease in Bcl-2/Bax protein ratio (**f**) and increase in the cleaved caspase-3 protein level (**g**) were blocked by a PI3K agonist 740 Y-P and a p38 MAPK antagonist SB203580, but were unaffected by a JNK antagonist SP600125. **P<0.01 *versus* control mice, ^#^*P*<0.05 and ^##^*P*<0.01 *versus* vehicle-treated GSK1016790A-injected mice

Among the three MAPK signaling pathways, the p38 MAPK and c-Jun N-terminal protein kinase (JNK) signaling pathways have been implicated in apoptosis in response to stress or some pathological conditions, such as cerebral ischemia.^14^ Modulation of the MAPK signaling pathway by TRPV4 activation has been previously reported.^7, 18^ Figures 2a and b show that an increase in phosphorylated p38 MAPK (p-p38 MAPK) protein level was found in the GSK-injected mice, whereas the protein level of phosphorylated JNK1/2 (p-JNK1/2) was nearly unchanged. These results indicate that activation of TRPV4 may enhance the activation of p38 MAPK signaling pathway.

Here, we examined the expression of Bcl-2 and Bax in the GSK-injected mice. Although the protein levels of Bcl-2 (Figure 2c) and Bax (Figure 2d) were higher in the GSK-injected mice, the Bcl-2/Bax protein ratio was significantly decreased after GSK1016790A treatment (*P*<0.01) (Figure 2f). In addition, the activation of caspase-3, which is an important mediator of apoptosis, was also assessed. As shown in Figure 2e, a significant increase in the cleaved caspase-3 protein level was found in the GSK-injected mice compared with that in the control mice (*P*<0.01). In our recent study, activation of TRPV4 has been shown to downregulate PI3K/Akt signaling pathway, which is involved in TRPV4-induced neurotoxicity.^7^ Here, it was determined that the decrease in Bcl-2/Bax protein ratio and the increase in the cleaved caspase-3 protein level were markedly rescued in the GSK-injected mice co-injected with 740 Y-P (a PI3K agonist) or SB203580 (a p38 MAPK inhibitor). The change in Bcl-2/Bax protein ratio or in the cleaved caspase-3 protein level in the GSK-injected mice was nearly unaffected by treatment with SP600125 (a JNK inhibitor) (Figures 2f and g). These results imply that activation of TRPV4 may negatively shift Bcl-2/Bax protein ratio and facilitate the activation of caspase-3 protein, which may be related to the inhibited PI3K/Akt and the increased p38 MAPK signaling pathways.

### Involvement of signaling pathways in TRPV4-induced apoptosis in hippocampus

**Figure 3:**
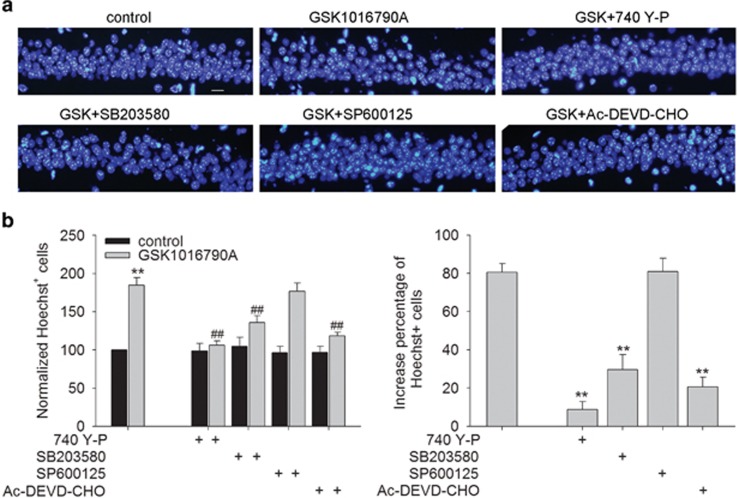
Involvement of signaling pathways in TRPV4-induced hippocampal apoptosis. Hoechst staining (**a**) and the bar graph (**b**) show that GSK1016790A-induced apoptosis in the hippocampal CA1 areas was attenuated by 740 Y-P (a PI3K agonist), SB203580 (a p38MPAK antagonist) or Ac-DEVD-CHO (a caspase-3 antagonist), but was unaffected by SP600125 (a JNK antagonist). **P<0.01 *versus* control mice, ^##^*P*<0.01 *versus* vehicle-treated GSK1016790A-injected mice

As shown in Figure 3, there were fewer Hoechst^+^ cells in the GSK-injected mice co-injected with 740 Y-P or SB203580 (*P*<0.01 in each case). Additionally, fewer Hoechst^+^ cells were found in the mice co-injected with GSK1016790A and Ac-DEVD-CHO (a caspase-3 inhibitor) (*P*<0.01). By contrast, the number of Hoechst^+^ cells in the GSK-injected mice was almost the same as that in the mice co-injected with GSK1016790A and SP600125. Combined with the above results, it is suggested that activation of TRPV4 may downregulate PI3K/Akt and upregulate p38 MAPK signaling pathways to decrease Bcl-2/Bax protein ratio subsequently and to activate caspase-3 at last, which is likely responsible for TRPV4-induced apoptosis.

### Effect of TRPV4 antagonist on brain infarction and changes in the protein levels of p-p38 MAPK, p-Akt and p-JNK at 48 h post MCAO

**Figure 4:**
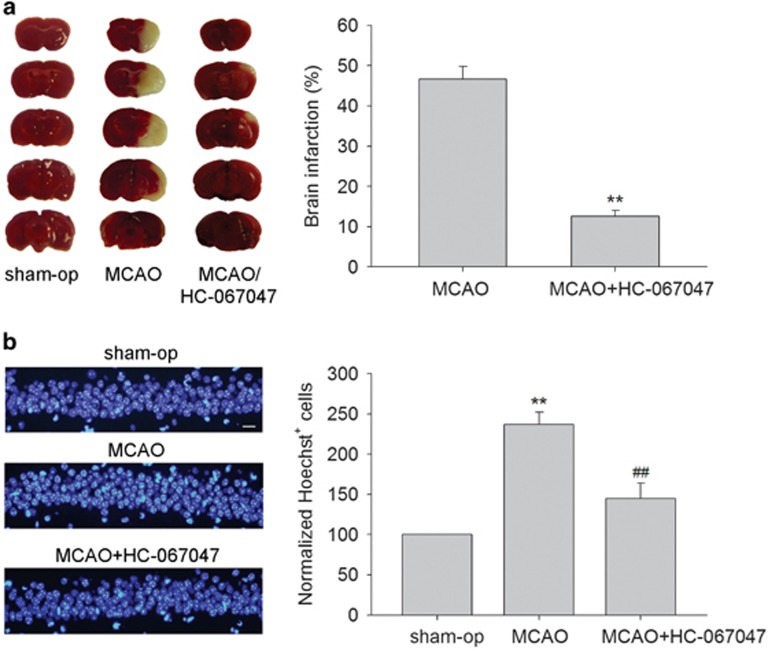
TRPV4 blockage-induced neuroprotection in MCAO mice. (**a**) Brain infarction at 48 h post MCAO was reduced by treatment with a TRPV4 antagonist HC-067047. **P<0.01 *versus* MCAO. (**b**) Hoechst staining shows that HC-067047 treatment reduced the number of Hoechst^+^ cells at 48 h post MCAO. Scale bar=50 *μ*M **P<0.01 *versus* sham-op, and ^##^*P*<0.01 *versus* MCAO

Cell apoptosis is evident at 24–72 h during ischemia-reperfusion.^12^ Here, we examined the effect of a TRPV4 antagonist on brain infarction at 48 h post MCAO. Figure 4a shows that the brain infarction volume was 46.67±3.14% in the MCAO mice at 48 h post MCAO. After the MCAO mice were treated with HC-067047, the brain infarction volume was only 12.59±1.46% (*P*<0.01). Here, more Hoechst^+^ cells were found at 48 h post MCAO (237.01±15.03%) and this change was markedly blocked following treatment with HC-067047 (145.11±19.21%) (*P*<0.01) (Figure 4b). Therefore, it is likely that the apoptosis caused by TRPV4 activation likely contributes, at least in part, to the neuronal injury on the condition of cerebral ischemia.

**Figure 5:**
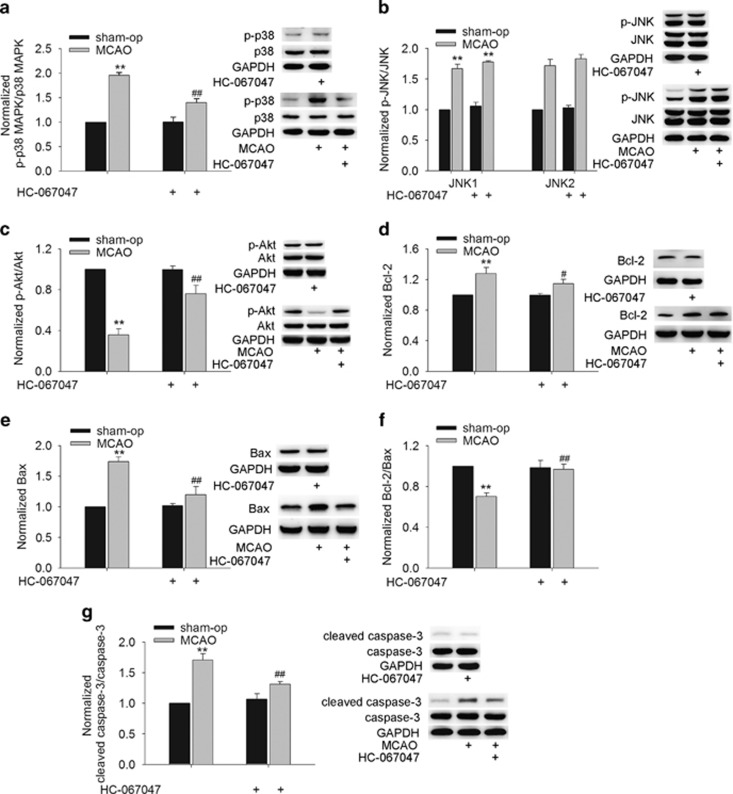
Effect of TRPV4 blockage on apoptosis-related signaling pathways and apoptosis-related proteins in MCAO mice. (**a**–**c**) The protein levels of p-p38 MAPK (**a**) and p-JNK1/2 (**b**) increased and that of p-Akt (**c**) decreased at 48 h post MCAO, but only the increase in p-p38 MAPK and the decrease in p-Akt protein levels were attenuated by HC-067047. (**d**–**f**) The increases in Bcl-2 (**d**) and Bax (**e**) protein levels at 48 h post MCAO were blocked by HC-067047. Note that the decrease in Bcl-2/Bax protein ratio (**f**) in the MCAO mice was markedly attenuated by HC-067047. (**g**) The increase in the cleaved caspase-3 protein level at 48 h post MCAO was markedly blocked by HC-067047. **P<0.01 *versus* sham-op, ^#^*P*<0.05 and ^##^*P*<0.01 *versus* MCAO

We also found that the protein levels of p-p38 MAPK (Figure 5a) and p-JNK (Figure 5b) were markedly increased and that of p-Akt (Figure 5c) was decreased significantly at 48 h post MCAO. Notably, after the MCAO mice were treated with HC-067047, the increase in p-p38 MAPK protein level and the decrease in p-Akt protein level were obviously rescued, whereas the increase in p-JNK protein level was nearly unaffected. Additionally, the decrease in Bcl-2/Bax protein ratio and the increase in the cleaved caspase-3 protein level in the MCAO mice were markedly attenuated by HC-067047 treatment (Figures 5d and g). These results indicate that during the cerebral ischemia, the activation of TRPV4 probably downregulates PI3K/Akt and upregulates p38 MAPK signaling pathways to decrease the Bcl-2/Bax protein ratio and activate caspase-3.

## Discussion

TRPV4 is sensitive to various types of stimuli, including hypoosmotic stimulation, mechanical force, the metabolism of AA and synthetic ligands. It is a calcium-permeable channel, and a TRPV4-induced increase in [Ca^2+^]_i_ has been confirmed by many research groups.^1, 2^ Increasing evidence of TRPV4 activation-related cellular toxicity is emerging. For example, sustained exposure to TRPV4 agonists has been shown to evoke the dose-dependent apoptosis of retinal ganglion cells, accompanied by an elevation in [Ca^2+^]_i_.^13^ TRPV4 has been demonstrated to be involved in human islet amyloid polypeptide-triggered apoptosis in a mouse pancreatic beta cell line and to be responsible for infrasound-induced apoptosis in the hippocampus.^5, 20^ A TRPV4 mutation displaying an increase in calcium channel activity results in increased cytotoxicity.^8, 21^ Consistent with our recent report concerning TRPV4-induced neuronal injury,^7^ the present data showed that application of a TRPV4 agonist dose-dependently induced apoptosis in the hippocampus (Figure 1), further demonstrating the neuronal cytotoxicity caused by TRPV4 activation.

Apoptosis is a type of programmed cell death, which is an initiative suicide process that occurs after cells receive a signal or stimulation. MAPKs are a family of serine/threonine protein kinases that are critical for the transduction of signals from the cell surface to the nucleus. Among the members of the MAPK family, extracellular signal-regulated protein kinase (ERK) is mainly responsible for the control of growth and differentiation, and the other two MAPK family members, JNK and p38, have roles in apoptosis as well as in inflammation, growth and differentiation.^22^ Akt has an important role in the suppression of apoptosis. After Akt is phosphorylated by PI3K, it can inhibit cell death by inactivating apoptogenic factors.^23^ It has been reported that Akt signaling pathway may be inhibited by the treatment with a TRPV4 agonist, and ERK signaling pathway may be activated by TRPV4 activation.^7, 18^ However, blockage of ERK pathway fails to attenuate TRPV4-induced hippocampal neuronal death.^7^ Therefore, the present study focused on exploring the involvement of Akt, JNK and p38 MAPK pathways in TRPV4-induced apoptosis. First, our data found that the protein level of p-p38 MAPK increased markedly, whereas that of p-JNK remained nearly unchanged in the GSK-injected mice (Figures 2a and b), indicating that activation of TRPV4 selectively activates p38 MAPK signaling pathway. Second, in the presence of a PI3K agonist (740 Y-P) or a p38 MAPK antagonist (SB203580), GSK1016790A-induced apoptosis was significantly attenuated. By contrast, the apoptosis caused by TRPV4 activation was unaffected by a JNK antagonist (SP600125) (Figure 3). These results indicate that the inhibition of PI3K/Akt and the increase in p38 MAPK signaling pathways are responsible for TRPV4 activation-induced apoptosis.

Bcl-2 family proteins are key regulators of apoptosis and include both anti-apoptotic members, such as Bcl-2, and pro-apoptotic members, such as Bax.^23, 24^ Here, the protein level of Bax was increased to a greater extent compared with that of Bcl-2 in the GSK-injected mice, leading to a decrease in Bcl-2/Bax protein ratio (Figures 2d and f). Caspases are a family of intracellular proteins that are involved in the initiation and execution of cell apoptosis. Caspase-3 is a potent, terminal caspase that executes apoptosis via a mitochondrial-dependent pathway.^25^ The present study showed an increase in the cleaved caspase-3 protein level in the GSK-injected mice, and fewer Hoechst^+^ cells were found in mice co-injected with GSK1016790A and caspase-3 inhibitor (Ac-DEVD-CHO), indicating that TRPV4-induced apoptosis is caspase-3 dependent (Figures 2e and 3). The above results indicate that activation of TRPV4 may result in a negative shift in the dynamic balance of the Bcl-2 family and ultimately activate caspase-3 that executes the apoptosis. Here, the decrease in Bcl-2/Bax protein ratio and the increase in the cleaved casapse-3 protein level in the GSK-injected mice were markedly attenuated by a PI3K agonist and a p38 MAPK antagonist (Figure 2g). Collectively, our data suggest that the over-activation of TRPV4 may inhibit PI3K/Akt and enhance p38 MAPK signaling pathways to negatively shift Bcl-2/Bax protein ratio and to ultimately activate caspase-3, which is probably involved in TRPV4-induced apoptosis.

There is increasing evidence that TRPV4 is a promising target for the treatment of cerebral ischemia. First, pathological changes that occur during ischemia/reperfusion, including cytotoxic cell swelling and disturbances in membrane lipid metabolism may facilitate the activation of TRPV4. Second, the protein level of TRPV4 increases with ongoing ischemia-reperfusion. Third, blocking TRPV4 has been proven to have protective effects on neurons following oxygen-glucose deprivation treatment or in rodent models of acute cerebral ischemia.^6, 7, 9, 10, 11^ Cell apoptosis is one of the major causes of cerebral ischemic injury, and it may be mediated through MAPK signaling pathways.^14^ The present study showed that application of a TRPV4 antagonist reduced brain infarction and apoptosis at 48 h post MCAO (Figure 4), indicating that TRPV4-induced apoptosis is likely involved in the cerebral ischemic injury. Here, the increase in p-p38 MAPK and the decrease in p-Akt protein levels in the MCAO mice were markedly rescued by HC-067047. Additionally, the decrease in Bcl-2/Bax protein ratio and the increase in the cleaved caspase-3 protein level in the MCAO mice were significantly inhibited following HC-067047 treatment (Figure 5). In summary, it is proposed that during the cerebral ischemia, the over-activation of TRPV4 results in caspase-3-dependent apoptosis through inhibiting PI3K/Akt and enhancing p38 MAPK signaling pathways, and this action contributes to cerebral ischemic neuronal injury.

The present study was a subsequent work of our previous report in which activation of TRPV4 shows neurotoxicity through enhancing the NR2B subunit of N-methyl-d-aspartate receptor and the related downregulation of Akt signaling pathway and thus is involved in the cerebral ischemic injury.^7^ In the previous study, we have proven that Akt but not ERK signaling pathway is selectively responsible for TRPV4-induced neuronal injury, providing a basis for further exploring the role of Akt signaling in TRPV4-induced apoptosis. In this study, p38 MAPK signaling was also confirmed to be responsible for TRPV4-induced apoptosis, whereas the involvement of JNK signaling was excluded. Furthermore, the inhibition of PI3K/Akt and the increase in p38 MAPK signalings were implicated in TRPV4-modulated apoptosis-related protein expression, including Bcl-2, Bax and cleaved caspase-3. In our previous study, brain infarction at 24 h post MCAO is reduced by TRPV4 antagonist HC-067047. Cell apoptosis becomes prominent after reperfusion for 24–72 h following cerebral ischemia;^12^ therefore, we examined the effect of HC-067047 on brain infarction and apoptosis at 48 h post MCAO in this study. Different time point post-MCAO chosen to study the effect of HC-067047 in cerebral ischemic injury was due to the different research objective in our previous study and the present one.

TRPV4 is widely expressed in the nervous system. Given its sensitivity to diverse stimuli and permeability to calcium, TRPV4 may have an important role in modulating the function of the nervous system under physiological and pathological conditions. In addition to its involvement in cerebral ischemic injury, the potential role of TRPV4-induced neurotoxicity has been reported in AD and motor neuron disease.^6, 8, 26^ An increase in TRPV4 expression has been found in the brains of aged rats.^3^ Therefore, targeting of TRPV4 provides a promising neuroprotective treatment.

## Materials and Methods

### Animals

Male mice (ICR, Oriental Bio Service Inc., Nanjing, China) weighing 25–30 g were used in this study. The animals were housed under a 12:12-h light/dark cycle and were provided food and water *ad libitum*. All animal experiments were approved by the animal experimental committee of Nanjing Medical University, China. Each experimental group contained nine mice.

### Drug treatment

All drugs were intracerebroventricularly injected as previously reported.^7^ After the mice were anesthetized with 2% chloral hydrate (20 ml/kg), they were placed in a stereotaxic device (Kopf Instruments, Tujunga, CA, USA). A 23-G stainless-steel guide cannula (Plastics One, Roanoke, VA, USA) was inserted into the right lateral ventricle (0.3 mm posterior, 1.0 mm lateral, and 2.5 mm ventral to the bregma) and anchored to the skull with four stainless steel screws and dental cement. Drugs were injected using a 26-G stainless-steel needle (Plastics One). The drugs were first dissolved in DMSO and then in 0.9% saline to a final volume of 2 *μ*l with a final DMSO concentration being 1%. SB203580 (1 mM/mouse), SP600125 (10 *μ*g/mouse), 740 Y-P (30 *μ*M/mouse) or Ac-DEVD-CHO (10 *μ*M/mouse) was injected 30 min before GSK1016790A injection and subsequently injected once daily for 3 days. The doses of the above antagonists or agonists were chosen as previously described.^10, 27, 28, 29, 30^ The TRPV4 antagonist HC-067047 was used as previously reported, except that it was repeatedly injected until 48 h post MCAO.^10^

### Preparation of the focal cerebral ischemia model

Transient focal cerebral ischemia was induced by MCAO as previously described.^7^ Briefly, after the mice were anesthetized, the right common carotid artery, external carotid artery (ECA) and internal carotid artery (ICA) were separated and exposed. The origin of the middle cerebral artery was occluded by a poly-l-lysine-coated nylon monofilament thread, which was inserted through the ECA and advanced into the ICA. Reperfusion was established by withdrawing the thread after 60 min of occlusion. The adequacy of vascular occlusion and reperfusion in the front parietal cortex of the occluded side was monitored using a multichannel laser Doppler flowmeter (Perimed PF5050, Jarfalla, Sweden). Body and head temperatures were thermostatically controlled and arterial blood pressure and gases were monitored through a femoral catheter during the operation. Mice in the sham-operated (sham-op) group were treated identically except for the occlusion of the middle cerebral artery.

### Infarction volume measurement

Brain infarction was determined using 2,3,5-triphenyl-tetrazolium chloride (TTC) staining as previously described.^10^ The brains were removed at 48 h post MCAO, sectioned into 2-mm-thick coronal slices and then incubated with a 2% TTC solution for 20 min. Brain infarction was visualized with image analysis software (MCID; Imaging Research, Canada), and infarct volume was determined as a percentage of the infarct area relative to the contralateral hemisphere area for each slice.

### Western blot analysis

Western blot analysis was performed at 48 h post MCAO or on day 3 after GSK1016790A injection. Hippocampal protein concentrations were determined with a BCA Protein Assay Kit (Pierce, Rochford, IL, USA). Equal amounts of protein were separated by SDS-polyacrylamide gel electrophoresis and transferred onto PVDF membranes. The membranes were blocked using nonfat milk in Tris-buffered saline (TBS)/Tween-20 and then incubated with antibodies against phospho-p38 MAPK (p-p38 MAPK, 1:1000, Cell Signaling Technology, Beverly, MA, USA), p38 MAPK (1:1000, Cell Signaling Technology, Boston, MA, USA), phospho-JNK1/2 (p-JNK1/2, 1:1000, Cell Signaling Technology, Boston, MA, USA), JNK1/2 (1:1000, Cell Signaling Technology, Boston, MA, USA), cleaved caspase-3 (1:1000, Cell Signaling Technology, Danvers, MA, USA), caspase-3 (1:1000, Cell Signaling Technology, Danvers, MA, USA), Bax (1:1000, Cell Signaling Technology, Boston, MA, USA), Bcl-2 (1:1000, Cell Signaling Technology, Boston, MA, USA) and glyceraldehyde-3-phosphate dehydrogenase (GAPDH) (1:5000, Abcam, Cambridge, MA, USA) at 4 °C overnight. After being washed with TBST, the membranes were incubated with an HRP-labeled secondary antibody, developed using an ECL Detection Kit (Amersham Biosciences, Piscataway, NJ, USA) and analyzed using Image J software (NIH). Hippocampal samples collected from the hemispheres of three mice were considered a set for western blot analysis. The summarized data represent the average of three experimental sets.

### Hoechst staining

On day 3 after GSK1016790A injection or at 48 h post MCAO, mice were anesthetized and perfused with ice-cold phosphate-buffered saline (PBS) followed by 4% paraformaldehyde. The brains were removed and immersed in fixative (4 °C overnight) and then processed for paraffin-embedding. Coronal sections (5 *μ*m) cut from the level of the hippocampus were stained with Hoechst-33342. Hoechst-positive (Hoechst^+^) cells were counted using a fluoresce microscope (Olympus PD70) ( × 40 objective). Hoechst^+^ cells were counted in six sections per mouse and expressed as the number of cells per millimeter of length along the hippocampal CA1 pyramidal layer.^19^

### Data analysis

Data are expressed as means±S.E.M. and were analyzed with Stata 7.0 software (STATA Corporation, College Station, TX, USA). ANOVA followed by Bonferroni's *post hoc* test was used for statistical analysis, and significance levels were set at *P*<0.05 and *P*<0.01. The number of the Hoechst^+^ cells or the protein level in the mice that were injected with GSK1016790A or/and kinase agonist/antagonist was expressed as a percentage of that in the vehicle-injected mice (control mice). The number of the Hoechst^+^ cells or the protein level in the MCAO mice or the MCAO mice treated with HC-067047 was expressed as a percentage of that in the sham-op mice. The increases in the number of Hoechst^+^ cells resulting from different doses of GSK1016790A were first normalized to the increase caused by 5 *μ*M GSK1016790A. The dose-response curve was then fitted by the Hill equation, in which *a*=*a*_max_/[1+(EC_50_/*C*)^*n*^], with *n* being the Hill coefficient, and EC_50_ being the dose of GSK1016790A producing 50% effect.

## Abbreviations

TRPV4: transient receptor potential vanilloid 4
MAPK: mitogen-activated protein kinase
ICV: intracerebroventricular
JNK: c-Jun N-terminal protein kinase
PI3K: phosphatidyl inositol 3 kinase
Akt: protein kinase B
MCAO: middle cerebral artery occlusion
TRP: transient receptor potential
AA: arachidonic acid
[Ca^2+^]_i_: intracellular free calcium concentration
ERK: extracellular signal-regulated protein kinase
ECA: external carotid artery
ICA: internal carotid artery
TTC: 2,3,5-triphenyl-tetrazolium chloride
GAPDH: glyceraldehyde-3-phosphate dehydrogenase
Hoechst^+^: Hoechst-positive
